# Liquid Biopsy in Pancreatic Cancer: Are We Ready to Apply It in the Clinical Practice?

**DOI:** 10.3390/cancers13081986

**Published:** 2021-04-20

**Authors:** Victoria Heredia-Soto, Nuria Rodríguez-Salas, Jaime Feliu

**Affiliations:** 1 Translational Oncology Research Laboratory, Biomedical Research Institute, La Paz University Hospital, IdiPAZ, Paseo de la Castellana 261, 28046 Madrid, Spain; victoriam.heredia@salud.madrid.org (V.H.-S.); nuria.rodriguez@salud.madrid.org (N.R.-S.); 2 Centro de Investigación Biomédica en Red de Cáncer, CIBERONC, Instituto de Salud Carlos III, Monforte de Lemos 5, 28029 Madrid, Spain; 3 Cátedra UAM-AMGEN, Medical Oncology Department, La Paz University Hospital, Paseo de la Castellana 261, 28046 Madrid, Spain

**Keywords:** pancreatic ductal adenocarcinoma, liquid biopsy, ctDNA, exosomes, CTCs, miRNAs

## Abstract

**Simple Summary:**

Pancreatic ductal adenocarcinoma (PDAC) is one of the tumors with the highest mortality, for which survival has hardly changed in the last 40 years. This high mortality is due to the lack of tests that would allow an early diagnosis and the fact that current treatments are not very effective. Liquid biopsy (LB) represents an interesting tool that can help in early diagnosis, treatment selection, disease monitoring, evaluation of the response and prognosis. It is a minimally invasive and risk-free procedure that can detect both the presence of genetic material from the tumor and circulating tumor cells (CTCs) in the blood and in other bodily fluids, and therefore distantly reflect the global status of the disease.

**Abstract:**

Pancreatic ductal adenocarcinoma (PDAC) exhibits the poorest prognosis of all solid tumors, with a 5-year survival of less than 10%. To improve the prognosis, it is necessary to advance in the development of tools that help us in the early diagnosis, treatment selection, disease monitoring, evaluation of the response and prognosis. Liquid biopsy (LB), in its different modalities, represents a particularly interesting tool for these purposes, since it is a minimally invasive and risk-free procedure that can detect both the presence of genetic material from the tumor and circulating tumor cells (CTCs) in the blood and therefore distantly reflect the global status of the disease. In this work we review the current status of the main LB modalities (ctDNA, exosomes, CTCs and cfRNAs) for detecting and monitoring PDAC.

## 1. Introduction

Pancreatic ductal adenocarcinoma (PDAC) represents the fourth leading cause of death from cancer in the world, with a 5-year survival of less than 10% [1,2]. In fact, unlike what happens with other tumors, its mortality has not been reduced in the last decades. Diverse estimates, based on the analysis of cases of the last 10 years, foresee an annual increase of 0.8% in its diagnosis, therefore, if there are no important advances, by 2030 it will represent the second cause of death from cancer in the United States and the EU, only below lung cancer [3,4,5].

Some of the factors that contribute to this high mortality are its insidious onset, with unspecific symptoms, and the lack of diagnostic tests and biomarkers that are sensitive and specific enough to allow its early detection. All this means that by the time of diagnosis 80% of tumors are already in incurable stages (locally advanced or metastatic). On the other hand, in patients with apparently localized disease, surgery is the only potentially curative treatment, but despite radical resection (R0), 80% of patients will present local or distant relapses after surgery [5].

To improve the current results of surgery, the therapeutic guidelines recommend administering adjuvant chemotherapy treatment for 6 months with the modified 5-fluorouracil-leucovorin-irinotecan-oxaliplatin (mFOLFIRINOX) regimen [6]. With this scheme, a median overall survival (OS) of 54.4 months and a median disease-free survival (DFS) of 21.6 months have been reported. However, this treatment can cause a high rate of grade 3–4 adverse events (affecting 76% of patients) [7]. This has led the guidelines to recommend its administration to patients with ECOG PS 0–1, leaving the combination of gemcitabine-capecitabine for patients with ECOG PS 2 [6]. On the other hand, despite the improvement that adjuvant treatment with mFOLFIRINOX supposes, 60% of the patients will still relapse after 3 years [7]. Therefore, to avoid exposure to unnecessary toxicity and optimize the efficacy of adjuvant treatment, it would be necessary to identify the patients who are really going to benefit from adjuvant treatment.

Neoadjuvant treatments (induction chemotherapy and/or chemoradiotherapy) followed by surgery are common for borderline-resectable PDAC and are under investigation in initially resectable disease. The results obtained suggest that this strategy facilitates complete resection (resectability), increases the probability of achieving negative resection margins, and improves OS [8,9,10,11]. In this context, the monitoring of the response to treatment is particularly relevant in order to determine the response of the tumor to preoperative treatment and therefore the resectability. However, various authors have pointed out that the radiological images, TC or MRI, obtained after neoadjuvant therapy do not predict with certainty the resectability of the tumor [11], so it is necessary to have more reliable methods of evaluation.

The standard of care for patients with locally advanced and metastatic PDAC is chemotherapy. Radiotherapy could be an option in locally advanced PDACs in which induction chemotherapy achieves control of the disease. Despite new chemotherapy regimens developed during the last years (FOLFIRINOX and gemcitabine plus nab-paclitaxel), the median OS continues under 12 months [12,13]. On the other hand, we do not have effective targeted therapies for most of the driver mutations detected in PDAC, although it has been reported that *BRCA1/2* mutations identify a small group of patients who benefit from platinum-based chemotherapy and PARP-inhibitors [14]. Likewise, the presence of microsatellites and *NTRK* gene fusions predict the response to immune checkpoints inhibitors [15] and tyrosine-kinase inhibitors [16], although the survival improvement is not as successful as the observed in other tumors. Despite all this advances, OS in PDAC has hardly improved in the last decades.

To improve the prognosis of patients with PDAC, it is essential to develop new treatments, but other strategies are also of interest, such as the identification of biomarkers that allow the personalization of treatments and help in early diagnosis, predict its recurrence and monitor the response to treatment and the evolution of the disease. Liquid biopsy (LB), in its different modalities, represents a particularly interesting tool for these purposes, since it is a minimally invasive and risk-free procedure that can detect both the presence of genetic material from the tumor and circulating tumor cells (CTCs) in the blood and other body fluids and therefore distantly reflect the global status of the disease [17,18].

**Figure 1:**
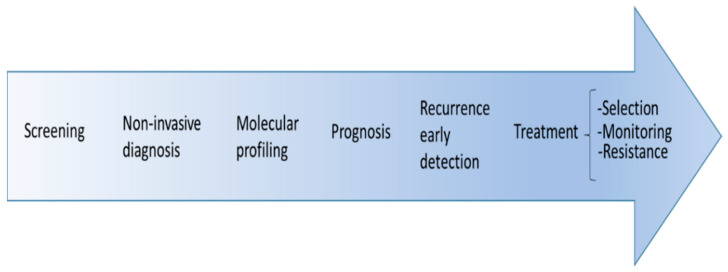
Potential application of liquid biopsy in pancreatic cancer.

An additional advantage of LB is that it avoids the traditional difficulty of obtaining a tumor sample in patients with advanced PDAC for molecular studies. In healthcare practice, the tumor sample is obtained using endoscopic-ultrasound guided fine-needle aspiration (US-FNA), which achieves a limited number of cells that, although sufficient for cytological diagnosis, often are not enough for molecular studies [19]. Furthermore, LB allows longitudinal monitoring of the molecular profile of the tumor during its evolution (Figure 1).

The objective of this work is to review the available evidence on the usefulness of the main LB modalities in PDAC.

## 2. Methodologies for Liquid Biopsy

**Figure 2:**
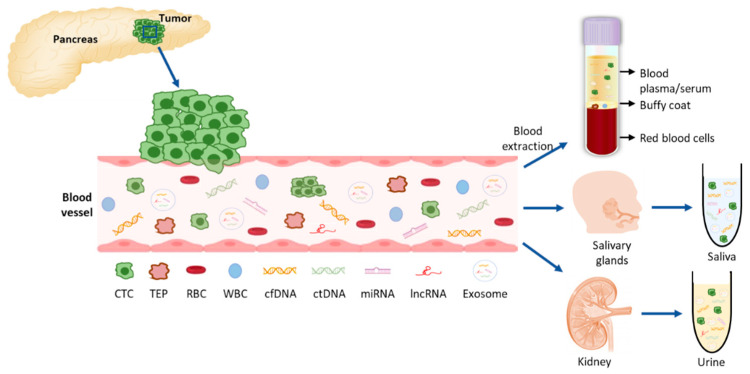
Liquid biopsy components in PDAC. Tumor cells (CTCs) are shedded from the tumor into the blood vessels where they can release their components: nucleic acids and exosomes with tumor-specific cargo material. For the analysis of these molecules, blood can be extracted and plasma or serum further processed for the extraction of the desired components. From the blood circulation, these molecules can be filtered to saliva and urine which can also be collected and further analyzed. CTC: circulating tumor cell; TEP: tumor educated platelet, RBC: red blood cell; WBC: white blood cell; cfDNA: cell-free DNA; ctDNA: circulating tumor DNA; miRNA: micro RNA; lncRNA: long non-coding RNA.

LB is a novel method for cancer diagnosis performed by analyzing and sampling of non-solid biological tissues, obtained primarily from blood, but also from other body fluids such as urine, saliva and cerebrospinal fluid [20]. Tumors and their metastases release biomarkers, mainly CTCs, cell free nucleic acids (cfDNA and cfRNAs), extracellular vesicles such as exosomes, and tumor educated platelets (TEPs), that can distantly reflect the disease (Figure 2). Therefore, liquid biopsies (LBs) represent a minimally invasive technique and allow diagnosis, real-time monitoring of cancer evolution and molecular follow-up of patients [21,22]. Also, LBs give us a better picture of the tumor heterogeneity than a tissue biopsy which only captures a specific area, since the whole tumor mass releases material into the blood [23].

Recent technological and molecular advances have increased our ability to detect and analyze LB components. In the following lines we will briefly introduce the different methods currently available for blood-based LBs (Table 1).

### 2.1. Cell Free DNA (cfDNA)

In cancer patients, cfDNA is released by tumor cells through apoptosis, necrosis, or active release. This DNA is called circulating tumor DNA (ctDNA) and it contains mutations that are specific to cancer cells [21]. cfDNA can be isolated from plasma or serum, but plasma is preferred to avoid possible contamination of serum with white blood cells during the clotting process [24].

ctDNA sequencing techniques need to be very sensitive and specific to overcome the low concentration of ctDNA in early stages of disease and the presence of cfDNA from normal cells that can result in false positives [23].

PCR-based sequencing methods are very sensitive, but they can only detect known variants. The most commonly used methods are real-time quantitative PCR (qPCR), which is fast and inexpensive but it can only detect mutant allele fractions (MAF) over 10%, and digital PCR (dPCR), an ultrasensitive detection method that separates the sample into thousands of parallel PCR reactions to reduce background noise and it can detect MAF inferior to 0.1% [23]. Droplet-dPCR (ddPCR) and BEAMing are the current standard approaches used for mutation detection in cfDNA with sensitivities between 0.01–0.1% [25].

Next-generation sequencing (NGS)-based methods permit high throughput analysis, can screen for unknown variants and can also identify structural variants and copy number variations, but with a lower sensitivity (approximately 1%) than dPCR. Platforms such as safe-sequencing system (Safe-SeqS) [26], Tagged-Amplicon deep sequencing (TAm-Seq) [27], Ion-AmpliSeq [28], CAncer Personalized Profiling by deep sequencing (CAPP-Seq) [29], and sensitive mutation detection using sequencing (SiMSen-seq) [30] have been developed. Recent advances have also enabled whole-genome sequencing in LB [23,24].

CtDNA methylation analysis has been used to evaluate cancer risk and monitor disease progression and metastasis. CtDNA methylation pattern is tissue and cell specific, and the information provided by these analyses can help determining the primary location of the tumor with high specificity and sensitivity [52,53]. Methylation-specific PCR [54] and MethyLight [55] platform have been used for this purpose and show a high sensitivity in the detection of these variants.

### 2.2. Exosomes

Exosomes are extracellular vesicles of endosomal origin secreted by all cell types that can be found in most body fluids. Their cargo material includes proteins, lipids, metabolites and nucleic acids (DNA and RNAs), and tumor derived exosomes (TDEs) contain specific surface proteins and other cellular contents that reflect the tumor cell of origin [22,56].

Methods for isolating exosomes can be density-based, size-based or affinity-based. Density-based separation is the most commonly used, and includes centrifugation or ultracentrifugation protocols that are time consuming, and the high speed of centrifugation might damage the exosomes and release contaminating proteins [20]. Size-based methods include size exclusion chromatography (SEC), a column-based technology that renders a purified exosomal fraction [31], filtration protocols with membranes of nanometer pore sizes, and microfluidic devices that use electrophoresis [32,33,34]. Both density and size-based methods result in contaminated products with low specificity and concentration of exosomes and cannot isolate TDEs. Affinity-based methods can isolate exosomes with higher purity and specificity, but result in a low sample yield. These approaches use antibody-coated magnetic beads or microfluidics that target proteins of interest such as exosome-specific tetraspanins [20,21].

Commercially available kits have been recently developed to facilitate exosome isolation with faster and simpler methods. Two of these kits are ExoTEST™ (Galen Laboratory Supplies, North Haven, CT, USA), an ELISA-based assay for quantitative and qualitative analysis of exosomes [22], and ExoQuick^®^ (SBI System Biosciences, Palo Alto, CA, USA), a proprietary polymer that gently precipitates exosomes and microvesicles [35].

### 2.3. Circulating Tumor Cells (CTCs)

CTCs are released from primary tumors and their metastases into the bloodstream and great advances have been made to achieve an efficient capture of these cells from whole blood. Current technologies include two main steps: CTC detection and CTC enrichment, and the techniques available are based on different methods that target either physical or biological/immunological characteristics of CTCs.

Immunoaffinity enrichment methods use specific antigens that are expressed on the surface of CTCs. When tumor cells are captured, the technique is classified as positive enrichment and epithelial cell adhesion molecule (EpCAM) and cytokeratin are the most common targets. When the target is an antigen that is not expressed on CTCs the technique is classified as negative enrichment, and CD45 is usually targeted for eliminating contaminating white blood cells [20,24]. These techniques are very specific, but a main drawback is that only one subpopulation of tumor cells can be captured, and tumor heterogeneity may not be represented unless multiple antibodies are employed. Also, recovering cells from the surface of the device may be difficult and cells can be damaged, a problem that is solved when using immunomagnetic strategies [36].

CellSearch^®^ (Menarini Silicon Biosystems Inc., Huntington Valley, PA, USA) is the current gold standard and the only US Food and Drug Administration-approved platform and uses magnetic beads covered with anti-EpCAM, anti-cytokeratin, and anti-CD45 to recover CTCs [20]. MACS^®^ (Miltenyi Biotec, Bergisch Gladbach, Germany) [37] and MagSweeper [38] are other platforms that use EpCAM-based markers, while AdnaTest and Strep-tag^®^ (Ibian Technologies, Zaragoza, Spain) use a cocktail of antibodies against multiple antigens (EpCAM, EGFR, and HER2) with a high capture efficiency and purity [24,39]. Other epithelial cells that have similar characteristics as epithelial CTCs may result in false-positive results and therefore epithelial-to-mesenchymal transition (EMT) and stem cell markers should also be considered [36].

Negative enrichment-based platforms using anti-CD45 antibodies are MACS^®^, Quadrupole Magnetic Sorter (QMS) [40], Dynabeads^®^ (Invitrogen, Carlsbad, CA, USA), and EasySep™ (Stemcell Technologies, Vancouver, Canada) [24]. These methods capture CTCs with a lower purity than positive enrichment methods, however, the advantage of negative enrichment methods is that label-free CTCs can be obtained [36].

Other immunocapture platforms that use positive enrichment affinity-based technologies are based on microfluidic devices such as CTC-chip [41], herringbone chip [42], high-throughput micro sampling unit (HTMSU) [43] and geometrically enhanced differential immunocapture (GEDI) chip [44]. These devices offer advantages for CTC research such as improved capture efficiency and high purity [24].

Physical methods rely on biophysical properties, mainly size and density. These methods are independent of antigen expression on the surface of a cell and better represent the tumor heterogeneity than antigen-based methods. Also, these methods are faster and simpler, they are less expensive and result in label-free, unmodified and viable cells, enabling subsequent downstream methods. However, these isolation methods must be followed with immunohistochemistry, immunocytochemistry, or immunofluorescence labelling to distinguish CTCs from other cell populations [20,36].

Size-based separation isolates CTCs depending on their increased size (9–19 μm). These methods mainly use membrane microfilters, with platforms as ISET^®^ (Rarecells Diagnostics, Paris, France) and ScreenCell^®^ (Sarcelles, France) and microfluidic sorting devices such as ApoStream^®^ (Precision for Medicine, Bethesda, MD, USA) and DEPArray™ (Menarini Silicon Biosystems), that use dielectrophoresis to separate CTCs [45,46]. Other microfluidic chips such as CTC-iChip and NanoVelcro, combine size and affinity to separate CTCs with higher purity and specificity [20,47,48].

Density-based methods use the specific density of red blood cells, leukocytes, and cancer cells to isolate CTCs by centrifugation gradients. Platforms that use this technology include OncoQuick^®^ (GrenierBioOne, Frickenhausen, Germany) and AccuCyte^®^ (RareCyte Inc., Seattle, WA, USA) [49,50], and can obtain recovery rates of 90% [36].

### 2.4. Cell-Free RNAs (cfRNAs)

RNA molecules have also been detected body fluids. Most studies are focused on non-coding RNAs, mainly microRNAs (miRNAs) and long non coding RNAs (lncRNAs). These RNAs are released by different cell components of the tumor and its microenvironment, mostly packed into exosomes or associated with protein complexes that prevent their degradation by RNases [57,58]. MiRNAs are short (19–23 nt), single-stranded non-coding RNAs, while lncRNAs are non-protein-coding transcripts with a length of more than 200 nt. They are both involved in the regulation of gene expression at the post-transcriptional level [59].

Sample quality and processing are important factors affecting the quality and quantity of cfRNAs. Pre-analytical and analytical aspects should be considered since physiological processes such as blood hemolysis of blood during plasma collection can influence the measurement of cfRNAs [60,61]. The methods used for the isolation of exosomes also can also affect the quality of exosomal miRNA profiles [51].

Many specialized kits for miRNAs isolation and a range of sample types, including serum and plasma, are commercially available and may be used with small sample volumes. lncRNAs can be isolated with regular RNA organic extraction methods [60,62,63]. Measurement and quantification of isolated RNAs rely on fluorometric methods such as Qubit, more sensitive and with a greater tolerance for the presence of contaminants than NanoDrop (spectrophotometry-based method), or Bioanalyzer (microfluidics-based automated electrophoresis) [60].

Methods for the evaluation of cfRNAs include sequencing and RT-qPCR. The main problem in cfRNA isolation from exosomes is their low concentration, which can be addressed with more sensitive techniques such as ddPCR [51]. Recently, PCR-free techniques have been developed such as electrochemical approaches [64] or thermophoretic sensors implemented with nanoflares for in situ detection of exosomal miRNAs [51,65].

### 2.5. Tumor Educated Platelets (TEPs)

It is now known that platelets can regulate tumor progression and metastasis as well as response to chemotherapy [66,67]. In the interaction between the tumor microenvironment and platelets, platelets uptake tumor proteins, extracellular vesicles and mRNA resulting in their education, and TEPs can then reflect the tumor-of-origin profile [22]. This interaction affects the expression of relevant genes in tumour cells and alters the RNA profile of the TEPs [68]. Studies have also shown that platelet number and size can provide information about the presence of cancer [69].

Platelets are a very abundant population in blood, so their isolation is relatively simple with a two-step centrifugation protocol. Whole blood can be stored for up to 48 h at room temperature prior to platelet isolation while maintaining high-quality RNA [22]. For their analysis, the thromboSeq platform has been developed, an RNA-sequencing-based methodology that enables identification of spliced RNA profiles from small amounts of platelet RNA [70].

## 3. Liquid Biopsy in Other Body Fluids for the Early Detection of PDAC

Molecular analyses for the early detection of PDAC via LB are also being developed in other body fluids such as pancreatic juice, saliva and urine. The collection of pancreatic juice from the duodenal lumen is less invasive than other tissue biopsy collection methods, but it is still a relative invasive and difficult technique that needs to be performed by specialized personnel. Nevertheless, a number of molecular studies have been performed for the diagnosis of PDAC using pancreatic juice, mainly for the detection of *KRAS* mutations [71]. A meta-analysis of 16 studies that analyzed the diagnostic value of *KRAS* mutations revealed that the sensitivity and specificity levels for the diagnosis of PDAC were 0.59 and 0.87, respectively [72], and another meta-analysis of 39 studies assessing the diagnostic value of the four major altered genes in PDAC (*KRAS*, *CDKN2A*, *TP53* and *SMAD4*), telomerase activity, and combination assay revealed that the most reliable marker was telomerase activity with a sensitivity and specificity of 0.82 and 0.96, respectively [73]. However, these results should be analyzed with caution since they cannot distinguish early PDAC from intraductal papillary mucinous neoplasm (IPMN), or pancreas with low-grade PanIN, since alterations of *KRAS* and telomerase activity are also found in these lesions [71].

Saliva is a very convenient fluid for LB determinations since it can be easily and noninvasively obtained from patients, and it has been reported that it contains almost the same molecules as serum because of the high blood flow in salivary glands [25]. Molecular studies have been performed in saliva for the diagnosis of PDAC. Exosomes have been found in saliva in preclinical models and have been reported to discriminate PDAC, hence, they might be potential biomarkers for detecting PDAC [74]. A salivary transcriptomic analysis has identified a four-messenger RNA panel (*MBD3L2*, *KRAS*, *ACRV1* and *DPM1*) that discriminates patients with PDAC from healthy controls with 0.9 sensitivity and 0.95 specificity [75]. MiRNAs miR-3679-5p and miR-940 have been reported to be down- and up-regulated in PDAC compared to healthy controls and benign lesions. The combined analysis of these miRNAs showed 0.7 sensitivity and 0.7 specificity in PDAC vs. noncancer [76]. The same group evaluated the expression of salivary lncRNAs and identified up-regulated levels of HOTAIR and PV1T in PDAC patients in comparison to healthy controls, with a combined sensitivity and specificity of 0.78 and 0.91 respectively. These values raised to 0.82 sensitivity and 0.95 specificity when differentiating PDAC from benign tumors [77].

Urine can be viewed as an ultrafiltrate of plasma and therefore may contain biomarkers that could assist with PDAC diagnosis [25]. Urine LB has the main advantages of allowing a completely non-invasive sampling and high volume collection, and has a lower proteome content than blood to avoid contamination of possible biomarkers [78]. Because of this, many metabolomic [79,80,81,82] and proteomic [83,84,85,86] studies have been conducted in order to identify possible biomarkers that can aid in the early identification of PDAC. With this purpose, Debernardi et al. have reported a urinary biomarker panel comprising LYVE1, REG1B, and TFF1 and PancRISK score that can discriminate patients with early stages of PDAC from control individuals and patients with benign hepatobiliary diseases [87].

Regarding cell free nucleic acids, detection of *KRAS* mutations in urine from PDAC patients has also been reported, and the detection rate and sensitivity are comparable to plasma LB [88]. Urinary miRNA biomarkers have also been analyzed and significant over-expression of miRNAs in PDAC Stage I versus healthy individuals (miR-143, miR-223, miR-30e) and Stage I versus Stages II-IV PDAC (miR-204, miR-143, miR-223) have been described [89]. A recent study also showed that the miR-3940-5p/miR-8069 ratio in urine exosomes is elevated in PDAC patients, suggesting that it may be a potential diagnostic tool for PDAC, especially in combination with CA19.9 [90].

## 4. Early Detection of PDAC

The only curative treatment for PDAC is surgery, and this is only possible when the tumor is diagnosed in early stages. However, in this phase of the disease, PDAC is usually a silent tumor and only in 10–20% of patients is it possible to make the diagnosis in early stages [91]. Currently we do not have sufficiently sensitive and specific tests to be used in the clinical practice, so no strategy has been established for early diagnosis in this tumor [92]. However, the 5-year survival of patients diagnosed in initial stages is 20%, while when there are metastases, it is only 3%, so it would be essential to have effective screening tests that allow increasing the proportion of patients diagnosed in early stages in order to improve survival. LB, in its different modalities, is being actively investigated in this field.

### 4.1. ctDNA

ctDNA represents less than 1% of all cfDNA. Its half-life is very short, since it is degraded by nucleases and eliminated through the urinary tract. Its concentration depends on the tumor volume, being detected more frequently in advanced tumors than in the early stages. All this makes its detection in early stages a technological challenge [93].

Mutations in *KRAS* represent the earliest genetic alteration driving PDAC and are found in over 90% of PDACs. It is possible to detect them in ctDNA, CTCs and exosomal DNA (exoDNA) [21], so their identification by LB could be very useful in the early diagnosis of PDAC. However, various studies have reported that the possibility of detecting ctDNA in patients with early stages is 30–65% [94,95,96,97] and 70–80% for locally advanced and metastatic PDACs [21] (Table 2).

A recent meta-analysis that included seven studies that evaluated the role of ctDNA in the diagnosis of PDAC found a pooled sensitivity of only 0.64 [113]. This relatively low sensitivity is attributed to the fact that in early PDAC the rate of necrosis and apoptosis is lower and not enough ctDNA is released into the peripheral blood [114]. In fact, it has been pointed out that in the initial stages of PDAC, only one ctDNA molecule can be detected for every 5 mL of plasma [113]. This represents a challenge that can be resolved as technology improves.

It has been recently reported that with dPCR techniques it is possible to detect *KRAS* mutations in up to 71% of patients with localized PDAC [99]. However, there is a risk that increasing the sensitivity of the technique may decrease its specificity. In fact, it has been suggested that it is possible to detect *KRAS* mutations in 7.4% of exoDNA and 14.8% of cfDNA in healthy controls [106], and in 20% of patients with chronic pancreatitis [115], and these numbers may increase as the sensitivity of the technique increases. It is unknown whether these positives may correspond to pancreatic pre-malignant processes or extra pancreatic *KRAS*-mutated tumors, and the possibility that driver mutations accumulate with aging has also been suggested [116].

It is also of interest to quantify ctDNA by determining the MAF. In fact, the MAF of *KRAS* in ctDNA is different in patients with pancreatic cysts, chronic pancreatitis, benign tumors, localized, locally advanced and metastatic PDAC [96,97,117].

The possibility of using ctDNA together with other biomarkers that could improve the sensitivity and specificity of LB in screening has been investigated. Thus, for example, the combination of *KRAS* mutations and CA19.9 showed a sensitivity of 0.98 and a specificity of 0.77 to differentiate PDAC from chronic pancreatitis [100]. Likewise, this combination of markers shows a sensitivity of 0.82 and a specificity of 0.81 to differentiate between PDAC and benign pancreatic tumors [96]. It has also been suggested that the combination of ctDNA with certain plasma proteins could improve the reliability of the test. In fact, in a study it was reported that the use of *KRAS* mutation in ctDNA allowed the detection of 30% of the cases, but the combination of *KRAS* mutation in ctDNA with four protein biomarkers (CEA, CA19.9, hepatocyte growth factor and osteopontin) achieved to detect 64% of patients. Furthermore, only 1/182 healthy controls were positive, giving a specificity of 0.99 [98]. This strategy is very promising for PDAC screening, although it needs to be validated in large population studies.

It has recently been suggested that epigenetic alterations -which are tissue- and cancer-type specific-potentially have a greater ability to detect and classify cancers in patients with early-stage disease. In a study that included several tumors, it was found that the analysis of cfDNA methylome allowed their early diagnosis [52]. In a pilot study it was reported that a model combining the modifications in 5-methylcytosine (5mC) and 5-hydroxymethylcytosine (5hmC) in cfDNA achieved a sensitivity of 0.94 and a specificity of 0.95, with an AUC of 0.99 for the diagnosis of PDAC [101].

A prospective case-control sub-study (from NCT02889978 and NCT03085888) assessed the performance of targeted methylation analysis of circulating cell-free DNA (cfDNA) to detect and localize multiple cancer types across all stages. 2482 cancer patients with more than 50 cancer types and 4207 non-cancer patients were included. Among the 84 patients included with PDAC in different stages, a sensitivity of 0.63 in stage I, 0.83 in stage II, 0.75 in stage III, and 1.0 in stage IV was found [118]. All these results suggest that the analysis of epigenetic modifications of cfDNA may be a very promising tool for the early diagnosis of PDAC.

### 4.2. Exosomes

Pancreatic cells have a strong exocrine function, which favors the continuous release of exosomes into the bloodstream. Furthermore, the half-life of exosomes is longer than that of ctDNA, so its detection does not depend on the occurrence of apoptosis or necrosis. These characteristics make them very interesting tools for early diagnosis [106]. Furthermore, exosomes carry the physiopathological signature of the cells from which they originate, both in membrane molecules and in the content of the vesicles [119]. For this reason, the analysis of their genetic material and that of their proteins can help in the early diagnosis of PDAC.

Although it was initially suggested that the presence of heparan sulfate proteoglycan glypican-1 (GPC1) could be detected in the exosome membrane of 100% of PDAC patients, and therefore could be used for the differential diagnosis with benign lesions [120], further studies found GPC1 in a much lower percentage, so that it did not allow its use for the diagnosis of PDAC [107].

Various authors have focused on the study of *KRAS* mutations in exoDNA. In a study that compared the performance of the determination of *KRAS* mutations in exoDNA and ctDNA, it was observed that they could be detected respectively in 66.7% and 45.5% of patients with localized PDAC, in 80% and 31% of locally advanced patients and in 85% and 58% of metastatic patients. However, these results could not be reproduced in the validation cohort, where it was only possible to find mutant *KRAS* exoDNA in 44% of early-stage PDAC patients [106].

Other studies have focused on the miRNAs of exosomes and have detected that PDAC patients present an increased expression of miR-17-5p and miR-21, which does not occur in healthy controls [108]. In another study, miRNAs miR-10b and miR-30c were found to be elevated and associated with the diagnosis of PDAC [107,109]. In addition, exosomal miRNAs would make it possible to differentiate pancreatitis from PDAC [107].

It has been reported that in the exosomes of patients with PDAC the pancreatic cancer-initiating cell protein CD44v6, tetraspanin-8, EpCAM and CD104 are increased and the miRNAs miR-1246, miR-4644, miR-3976 and miR-4306 are overexpressed, while this does not occur in healthy controls, which opens the possibility of using these molecules as biomarkers for early diagnosis [110].

### 4.3. CTCs

CTCs are tumor cells that invade blood vessels during the metastasis process. They can be found as isolated cells or as cell clusters forming a tumor microthrombus. Various studies have indicated that CTCs can be detected in 21–100% of patients with PDAC, depending on the series, stage, and technique used [113,121,122]. However, compared to other epithelial tumors, fewer CTCs are detected in PDAC and in fewer patients, so the sensitivity of this technique for early diagnosis is low.

Despite its variable sensitivity, its specificity is very high, since they are hardly found in healthy controls. In a study with early-stage tumors, CTCs could be found in 75–80% of patients [123,124], but other studies have reported detection rates below 50% [102]. Furthermore, it has been reported that it was possible to find circulating epithelial cells (CECs) in 33% of patients with intraductal papillary mucinous neoplasm (IPMN) without PDAC, and in 73% of PDACs [102]. Similarly, in another study the same proportion of CECs was detected in patients with benign, premalignant, and malignant lesions, although they were not found in healthy controls [125].

In a meta-analysis that included 19 studies with a total of 1872 patients, it was observed that the sensitivity, specificity and AUC for the diagnosis of PDAC when using ctDNA were 0.64, 0.92 and 0.94, if exosomes were used they were 0.93, 0.92 and 0.98 and if the CTCs were studied they were 0.74, 0.83 and 0.81 respectively [113]. This lower AUC of CTCs compared to other LB modalities can be attributed to the fact that CTCs can become trapped in the liver when they travel through the portal vein, and because, compared to normal pancreatic tissue, the blood flow within the pancreatic tumor tissue is reduced by 60% [113]. In fact, it has been pointed out that when blood from the portal vein is analyzed, the ability to detect CTCs notably increases, therefore, they can be found in 100% of patients with metastatic PDAC [126] and in 58% of resectable patients [104]. The problem is that this type of procedure increases the invasiveness and complexity of the technique.

The ability to detect CTCs not only depends on the stage of the PDAC, but also on the technology used. A recent study suggests that combining the determination of CTCs by two different procedures (RosetteSep^TM^ and CellSearch^®^ (Menarini Silicon Biosystems)) together with the determination of positive GPC1 exosomes increases the sensitivity of the procedure up to 100%, although with a specificity of 80%, since they could identify CTCs in 50% of the IPMN and in 10% of the healthy controls [105] (Table 2).

### 4.4. cfRNAs

The possible role of plasma miRNAs for the early diagnosis of PDAC has also been investigated. Various miRNAs that could be used as biomarkers in this scenario have been identified, among which miR-21 and miR-25 stand out. The results of a meta-analysis showed that miR-21 would have a sensitivity for early diagnosis of 0.90 and a specificity of 0.72 [127]. In another study miR-25 was also reported to have a high capacity for PDAC diagnosis with a sensitivity of 0.75 and 0.93 specificity [111].

On the other hand, it has been suggested that the combination of several miRNAs could improve the diagnostic capacity. It has recently been proposed that a signature made up of 13 miRNAs would make it possible to distinguish PDAC patients from healthy subjects [128].

Circulating miRNAs can also aid in the differential diagnosis between benign and malignant IPMN. It has been reported that miR-233 is not only elevated in the plasma of patients diagnosed with PDAC, but is also found in higher levels in malignant than benign IPMN [129]. Likewise, miR-196a and miR-196b are higher in patients with PanIN2-3 than in patients with PanIN-1 [130].

In patients at high risk of developing PDAC or with a history of familial pancreatic cancer, it was observed that a panel composed of miR-196b, LCN2 and TIMP1 allowed the differentiation of high-grade lesions and stage I PDACs from healthy controls [131].

A recent meta-analysis, which included a total of 46 studies, 4326 PDAC patients and 4277 non-PDAC controls, reported that miRNAs offered a cumulative sensitivity for the diagnosis of early stage PDAC of 0.79 and a specificity of 0.74. Furthermore, they found that the combination of miRNAs and CA19.9 could improve these figures [112].

We can conclude by stating that the studies carried out for the early diagnosis of PDAC with LB are promising, although the sensitivity and specificity of the different techniques must be improved. Probably the combination of some of them together with certain biomarkers will improve their reliability. In any case, before it can be applied in healthcare practice, it is necessary to verify its performance in clinical trials with an adequate number of patients. Ongoing clinical trials that use LB in PDAC are shown in Table 3.

lncRNAs are also involved in cellular processes in PDAC, such as cell proliferation, epithelial-mesenchymal transition (EMT) and metabolic reprogramming [132]. Abnormal expression of lncRNAs has also been identified in pancreatic cancer and may be involved in cell growth, apoptosis, invasion, metastasis and angiogenesis [25]. Promising results have been reported in pre-clinical studies that point at these molecules as possible diagnostic and prognostic biomarkers.

The expression levels of lncRNA SNHG15 were studied in a series of 171 PDAC patients, and the results showed that SNHG15 was upregulated in PDAC tissue and serum samples compared with paracancerous tissue and healthy controls, so, SNHG15 may be a potential biomarker for differentiating PDAC tissues from normal pancreatic tissues. Clinicopathologic analysis revealed that high SNHG15 expression was associated with tumor differentiation, lymph node metastasis and tumor stage (*p* < 0.005), and patients with high SNHG15 expression had a shorter OS compared with the low SNHG15 expression group (*p* = 0.003). Also, Cox multivariate analyses confirmed that SNHG15 expression was an independent prognostic factor in PDAC (*p* < 0.004) [63].

Other lncRNAs such as POU6F2-AS2 or extracellular vesicle-encapsulated HULC have been found in higher levels in the sera of patients with PDAC than in healthy controls and could be explored as potential biomarkers for human PDAC [133,134].

Yu et al. have developed a d-signature with eight long RNAs from plasma extracellular vesicles for PDAC detection in a study that enrolled 501 patients. This d-signature is able to identify resectable stage I/II cancer with an AUC of 0.949 and shows a better performance to CA19.9 in distinguishing PDAC from chronic pancreatitis (AUC 0.931 vs. 0.873, *p* = 0.028) [62].

### 4.5. Liquid Biopsy in the Diagnosis of PDAC

Currently, the diagnosis of PDAC is made by imaging tests and obtaining tumor samples, usually by ultrasound-guided fine needle aspiration (US-FNA). This test is invasive, and either due to technical difficulties or due to the important stromal component of the tumor, tumor cells are sometimes not obtained to confirm the diagnosis, even after repeating the test. In these cases, surgery is necessary to obtain a tumor sample. In this context, LB could help confirm the diagnosis without subjecting the patient to the risks of aggressive testing. However, the performance of LB varies with the tumor stage, so that in patients with resectable disease, ctDNA is detected less frequently than in patients with unresectable disease and, in addition, they present fewer genomic alterations [129].

A study that compared the diagnostic value of CTCs, ctDNA and CA19.9, showed that while the sensitivity and specificity for PDAC diagnosis for US-FNA were 0.73 and 0.88, for ctDNA were 0.65 and 0.75, for CTCs were 0.67 and 0.80, and 0.79 and 0.93 for CA19.9, respectively. In patients with positivity for two of these biomarkers, sensitivity and specificity improved, reaching 0.78 and 0.91, respectively [94]. A recent meta-analysis demonstrated that in patients with advanced disease, the sensitivity and specificity for PDAC diagnosis of ctDNA were 0.64 and 0.92, for exosomes 0.93 and 0.92, and for CTCs 0.74 and 0.96, respectively [113].

## 5. Detection of Recurrence

In PDAC patients, recurrences after surgery are very frequent. In resectable PDAC they are detected in up to 60% of patients during the three years following surgery [7], and in borderline cases in 80% of patients in the first year after the surgical process [135]. The conventional prognostic factors of PDAC are usually tumor size, lymph node status, perineural invasion, and resection margin, but they have the disadvantage that they can only be obtained after surgery. However, various evidences suggest that LB can offer prognostic information from the moment of diagnosis, but also during follow-up and tumor progression. After completing surgical treatment and adjuvant treatment, a follow-up program is usually started that includes serial assessment using CT imaging and CA19.9 testing. However, various meta-analyses indicate that CT and CA19.9 only have moderate diagnostic accuracy for the diagnosis of recurrent PDAC [136,137]. It should also be noted that CA19.9 is limited by its low sensitivity and that 5–7% of patients have Lewis-negative genotypes and, therefore, are unable to produce CA19.9 [138]. In this context, the possible role of the different LB modalities to detect recurrence is being investigated.

### 5.1. ctDNA

Since the half-life of ctDNA is very short, after complete removal of the tumor, no ctDNA should be detected. However, its persistence after surgery indicates that the disease has probably remained and, therefore, there is a high risk of recurrence.

Various studies have indicated that the determination of *KRAS* mutated ctDNA during follow-up after surgery has a sensitivity and specificity of around 0.90 to predict recurrence, anticipating its detection by imaging techniques by several months [135,139]. After surgery, it is possible to detect ctDNA in 30–45% of patients [95,139], and in these cases, the relapse rate is very high, reaching 100% in some series [95]. In addition, the median Relapse Free Survival (RFS) ranges from 5–10 months. These data suggest that in patients in whom ctDNA is detected after surgery, adjuvant treatment should be administered and ctDNA levels should be monitored, since its elevation during treatment could be an indicator that resistance has developed and, therefore, modification of treatment should be considered.

Likewise, the detection of *KRAS* mutated ctDNA before surgery implies a poor prognosis, so that it predicts both decreased median RFS (6 vs. 16 months; *p* < 0.001) and decreased OS (14 vs. 28 months; *p* < 0.0001) [140]. In one study, which included patients with resectable PDAC, *KRAS* mutated ctDNA was detected in 62% of patients before surgery, and it was associated with significantly lower RFS and OS. In fact, 83% of patients with detectable ctDNA at diagnosis relapsed [95]. These data suggest that those patients with resectable PDAC in whom ctDNA is detected before surgery are a high-risk group in which neoadjuvant therapy strategies could be explored. When ctDNA was detected at diagnosis and became negative after surgery, the risk of relapse was lower than when ctDNA levels remained high [95].

In addition to the detection of ctDNA, it has been pointed out that its quantification also has prognostic importance, since patients with an elevated MAF prior to surgery have a higher risk of relapse and a lower DFS [99].

### 5.2. Exosomes

TDEs may reflect the response to surgery, such that their persistence after resection is related to the presence of hidden metastases. Patients with more than 20% GPC1 positive exosomes in peripheral blood have been reported to have lower progression-free survival (PFS) and OS [105]. Similarly, the detection of high exosome-encapsulated miR-415a was also associated with worse PFS or OS [141]. Furthermore, the detection of exosome-encapsulated miR-4525, miR-415a, and miR-21 in the portal vein was more sensitive in identifying patients at high risk of recurrence after surgery than its detection in peripheral blood [142].

### 5.3. CTCs

In patients with resectable PDAC, it is possible to detect CTCs in percentages ranging between 5 and 60% of cases depending on the technique used [143,144,145]. The detection rate with CellSearch^®^ has been reported to be 26% (95% CI: 14–38%), which is lower than the figures obtained with filtration or microfluidic techniques. In addition, the detection rate is significantly increased when blood from the portal vein is analyzed compared to peripheral blood [146]. It has been reported that 85% of patients with CTCs in the portal circulation developed liver metastases, while this only occurred in 13% of patients in whom they were not detected [104].

Both preoperative chemotherapy and surgery achieve a significant reduction in CTCs, but not their complete disappearance. In the three days following surgery, a decrease in CTCs is observed, and their subsequent increase may indicate the existence of metastatic disease [121]. Recurrences have been observed to occur more frequently in patients in whom CTCs are detected than in those in whom they are not [147].

In addition to the presence and number of CTCs, the type of CTC is also of prognostic importance [144]. Two subtypes of CTCs have been identified in PDAC patients: epithelial and epithelial/ mesenchymal, so that the detection of CTCs with positive stain for cytokeratin (epithelial marker) is associated with worse survival than if CTCs are positively stained with vimentin (mesenchymal marker) [144]. Furthermore, the detection of CTCs that co-express cytokeratin and vimentin is associated with a shorter relapse-free time [147]. Likewise, the detection of a tumor initiating cell (TIC) phenotype (evaluated with the expression of aldehyde dehydrogenase, CD133, and CD44) was associated with tumor recurrence and decreased DFS [145]. In another study, it was reported that the detection of cancer stem cells expressing ALCAM, CD44, and POU5F1B was related to more aggressive tumors and a worse prognosis [148,149].

### 5.4. miRNAs

In surgical patients, elevated levels of miR-744 and downregulated levels of miR-373-3p are associated with a poor prognosis, the appearance of recurrences and the development of metastases, and resistance to chemotherapy [150]. Increased expression of miR-18a has also been related to recurrence after surgery, and this elevation may be independent of the CA19.9 level. The same occurs with the elevation of miR-21-5p [151]. On the contrary, the decrease in miR-196a, miR-196b, miR-221 and miR-483 after surgery would be related to a favorable prognosis [152,153].

The results of these studies suggest that LB in its different modalities can help in an early detection of PDAC recurrence after surgery. A positive LB after surgery suggests the presence of viable tumor tissue and, therefore, the necessary tests should be carried out to locate the residual disease in case its eradication is possible or, alternatively, start a treatment to delay the progression of the tumor. An unresolved question is whether the possible clearance of ctDNA after adjuvant treatment implies an improvement in the prognosis. This question is intended to be answered with the DYNAMICS-Pancreas clinical trial that investigates the benefit of intensifying adjuvant treatment in those patients in whom ctDNA continues to be detected after surgery (Table 3).

On the other hand, in patients with undetectable ctDNA after surgery it may be considered not to administer adjuvant treatment. Unlike what occurs in other tumors such as colon cancer, in PDAC the few data available suggest that these patients continue to relapse in a significant percentage, although less than that of those with detectable ctDNA. Alternatively, a less aggressive or shorter adjuvant treatment could be considered, but the safety of these strategies needs to be demonstrated through prospective clinical trials.

## 6. Disease Monitoring

Current chemotherapy treatments have limited efficacy and yet can cause significant toxicity. For this reason, it is important to rapidly identify patients who do not benefit from treatment in order to suspend it and, if possible, start a new therapy. LB could help in the early detection of response to treatment and tumor progression, and thus guide treatment decisions. There are at least two situations where disease monitoring acquires special relevance: in the context of neoadjuvant treatment and in metastatic disease. The utility of LB has been investigated in both settings (Table 4).

### 6.1. Neoadyuvant Treatment.

One of the great challenges of oncology is the detection of minimal residual disease (MRD) after neoadjuvant treatment. Chemotherapy with or without radiotherapy can achieve pathological complete response (pCR) in 3–11% of patients diagnosed with PDAC [161]. In these cases, the prognosis notably improves, and as in other neoplasms, if it were certain that the entire tumor had been completely reduced, it could be speculated on the possibility of avoiding the inconveniences of a complex surgical procedure, which can cause a high morbidity and even mortality. Likewise, it is of interest to monitor the disease after surgery, in order to verify, in the event that MRD remains, that adjuvant treatment manages to eliminate it.

Despite the better prognosis accomplished by achieving a pCR, more than half of the patients will relapse after surgery. In fact, a study that analyzed the presence of ctDNA and CTCs after surgery in patients who had achieved pCR after neoadjuvant treatment, reported that it was possible to detect CTCs in 5 of 6 patients and ctDNA in 7 of 16 patients with pCR. This led the authors to propose the concept of molecular complete response (mCR) that would combine the study of the biopsy with the genomic analysis of the resected tissue [162].

In a study that included 59 patients, of whom 30 underwent surgery directly and 20 received neoadjuvant treatment, ctDNA could be detected before surgery in 69% of those who underwent surgery without prior neoadjuvant therapy compared to 21% of patients who received neoadjuvant treatment. The presence of preoperative ctDNA was associated with a significantly lower recurrence-free survival (RFS) and OS. In addition, all the patients in whom ctDNA was still detected after neoadjuvant treatment relapsed, with a median RFS of 5 months [135].

It has been proposed that monitoring of *KRAS* MAF in exoDNA during neoadjuvant treatment can help predict PDAC resectability. In fact, in a study it was observed that 71% of patients in whom PDAC could be resected had a decreased *KRAS* MAF, while in 94% of patients whose PDAC was not resectable after neoadjuvant treatment, *KRAS* MAF remained stable or increased [94]. Furthermore, the increase in exoDNA after neoadjuvant treatment was significantly associated with disease progression [97].

### 6.2. Metastatic Disease

Various studies have shown that ctDNA can be detected in the plasma of 70% of patients with metastatic PDAC, and that its presence is associated with a worse prognosis. Thus, for example, in a series of 104 patients, the median OS of patients in whom ctDNA could be found was 6.5 months compared to 19 months in those without detectable ctDNA [154]. The results of other studies have confirmed the negative prognosis of ctDNA detection in patients with locally advanced or metastatic PDAC [97,155,156,157].

The presence of ctDNA, in addition to serving for prognosis, can be used as a biomarker to monitor the response to treatment and indicate early resistance to it, so that treatment can be modified depending on the evolution of the disease. In a study with 54 patients with advanced PDAC undergoing chemotherapy, increased *KRAS* mutant ctDNA predicted disease progression with a sensitivity of 83% and a specificity of 100%. Furthermore, its decrease during treatment can predict its response [158]. In another study, negative ctDNA during chemotherapy was associated with a higher PFS than in those patients in which it was still detected [163], although this extreme could not be confirmed by other authors [97]. In a phase II clinical trial conducted in patients treated with second line chemotherapy, the prognostic and predictive value of ctDNA was investigated. 113 patients were included, and ctDNA was detected in 77% of them. It was observed that patients with detectable ctDNA had worse PFS and OS, but also, an early change in ctDNA levels was correlated with a higher response rate and a significant increase in PFS and OS [159].

In a study that included 13 patients with metastatic PDAC and studied the ctDNA mutations of several genes such as *KRAS*, *BRCA2*, and *EGFR,* it was observed that new mutations in ctDNA could be detected in patients progressing to chemotherapy [160].

PDAC has a large stromal component, so radiological images often do not adequately reflect the response to treatment. In this situation, LB may also be helpful. In fact, the previous study also investigated the concordance in the evaluation of the response by CT and by ctDNA, and observed that although in 10 patients (77%) the result of the evaluation coincided with the two techniques, in 60% of the cases ctDNA provided the earliest measure of treatment [160].

The optimal time to perform the LB procedure is unknown. It has been observed that 7 days after starting treatment there may be elevations that lack prognostic significance, but variations at 14 days may anticipate a radiological response [149].

Several authors have reported that the dynamic changes experienced by ctDNA during treatment are not only related to the evolution of tumor volume, but also to PFS and OS, so that it can serve as an early marker of response and as prognostic factor [117,158,161]. Furthermore, increases in ctDNA MAF during treatment are associated with lower PFS [117].

Applying NGS techniques to the ctDNA of 188 patients with metastatic PDAC, mutations in *KRAS* G12V and *ERBB2* exon 17 were reported to be associated with reduced survival. Furthermore, mutations in *KRAS* G12 were related to radiological responses [160]. On the other hand, it has been pointed out that higher methylation index values for *SPARC* and *NPTX2* genes in cfDNA were found to associate with poor survival in patients with PDAC [164].

In patients with advanced PDAC, CTCs can be detected in a percentage that varies depending on the technique used and the stage of the disease. Thus, for example, while some authors found CTCs in 11% of patients with locally advanced PDAC [165], others, using antibody-independent CTC isolation, have reported its detection in 77% of patients with locally advanced stages and in 93% of stage IV [166]. Even with the use of the CTC-Chip, its detection has been reported in 100% of patients [41].

In a meta-analysis that included nine studies with a total of 600 patients with any stage PDAC, CTCs were identified in 43% of the patients, and it was observed that their PFS and OS were significantly lower than those in which they could not be detected [167].

In addition to its prognostic value, the determination of CTCs during treatment can guide the response to chemotherapy. It has been observed that after a chemotherapy cycle with 5-FU, the percentage of patients in whom more than 2 CTCs could be detected decreased from 80% to 29% [123]. In a study with 40 patients, dynamic changes in CTCs were investigated during chemotherapy treatment, and CTCs were observed in 45% of patients with progression or stable disease and only in 24% of those who achieved partial response. Detection of CTCs was associated with a worse prognosis [168].

It has been reported that when the tumor progresses after chemotherapy, the CTCs population also changes, with an enrichment in the CTCs of the expression levels of stemness and pluripotency genes such as *CD44*, *ALCAM*, *EPCAM*, *NOTCH1*, *POU5FIB*, and *PTCH1*, or CSC drivers as *VEGFB* and *STAT3* [149]. Likewise, it has been proposed that CTCs could serve to monitor the response to treatment, and indicate early resistance to it [169]. Furthermore, it has been suggested that the pharmacogenomic study of RNA extracted from CTCs could help predict the response to PDAC treatment and help optimize treatment [170].

A study reported that the presence of MUC-1, a large trans-membrane glycoprotein in the CTCs of PDAC patients was associated with a shorter OS than those with MUC-1 negative CTCs (2.7 vs. 9.6 months). Furthermore, the presence of anti-MUC-1 antibodies was associated with longer survival [171]. In another study, the type of *KRAS* mutation was found to have prognostic value, such that those with the *KRAS* G12V mutation had a better prognosis than those with other or lacking *KRAS* mutations [172].

## 7. Precision Medicine

ctDNA can provide clinicians with information on PDAC mutations and on the epigenetic characteristics of the neoplasm [173,174]. There are important discrepancies between the mutations detected in tumor and in plasma. In a study that included PDAC and colorectal cancer patients and studied 56 genes by NGS in tumor tissue and in plasma, it was observed that 78% of the mutations detected in plasma were not found in the primary tumor [175]. In a recent meta-analysis, it was observed that considering studies that analyzed *KRAS* mutations only, the concordance was 65%, but when considering all mutations detected by multi-genes panels the concordance was only 31.9% [176].

These discrepancies can be attributed to the fact that the mutations present in the primary tumor can be detected in the ctDNA, while in the analyzed tissue, due to tumor heterogeneity, there may be mutations that are not detected [175]. On the other hand, the presence of different clones in the metastases could also contribute to these discrepancies. In fact, some studies have shown that driver gene mutations in PDAC are usually maintained during clonal evolution [177,178], but throughout the progression of cancer, other molecular alterations are added that are detectable in the metastases but not in the primary tumor [175,178].

LB describes in a more complex manner real time dynamics of tumor disease, providing complete information on the tumor genome, and enables the identification of changes that occur during tumor treatment [174].

PDAC is associated with alterations in driver genes such as *KRAS*, *CDKN2A*, *ERB2*, *BRCA1/2*, *NTRK*, etc., and these tumors are susceptible to targeted treatments. In a recent retrospective study that included 1856 PDAC patients, 1082 (58%) received personalized reports based on their molecular testing results, and actionable molecular alterations were identified in 26% of the patients. Of these, 46 patients received matched therapy and had a significantly higher OS than those who received unmatched therapies (2.58 years vs. 1.51 years, *p* < 0.0001) [179].

In another study, a potentially actionable mutation was detected in 29% of patients, including *ALK* (*ALK* inhibitors), *ATM* (DNA cross-linking drugs or poly (ADP-ribose) polymerase inhibitors), *DNMT3A* (*DNMT* inhibitors), *EGFR* (EGFR inhibitors), *KIT* (KIT inhibitors), *MAP2K4* (*MEK* inhibitors), and *PIK3CA* (*PI3K/AKT/mTOR*) pathway inhibitors [180]. In another study, a panel of 73 genes analyzed 357 samples from 282 patients with advanced PDAC. After excluding variants of unknown significance, therapeutically relevant alterations were observed in 170 (48%) samples. There were 40 patients in whom a ctDNA sample taken at the time of diagnosis and progression was available, and it was observed that in 23 (57%), ctDNA analysis allowed the detection of new genetic alterations at disease progression. None of these newly acquired genetic alterations were identified on tissue profiling performed at diagnosis [181].

These results highlight the importance of molecular studies in a tumor where the therapeutic options are very limited and the finding of an actionable mutation represents a great therapeutic opportunity. In addition, given that new genetic alterations may appear with tumor progression, it is important to monitor these molecular changes throughout the evolution of the disease. Given the difficulties that exist in obtaining a tumor sample in PDAC, LB, due to its simplicity, accessibility, and the importance of the information it can provide, should play a relevant role. In this sense, it should be noted that techniques are being investigated to study the molecular profile and gene expression of CTCs, which would allow us to know the gene expression changes that occur in tumor cells during treatment and the possible development of resistance [182].

In any case, for the use of LB to spread in healthcare practice, it is necessary to define the most appropriate technique, reduce costs, and have the results of prospective studies that support the suitability of this tool.

## 8. Conclusions

Although there were many expectations set on the use of LB for early diagnosis, the truth is that the relatively low sensitivity and specificity of current techniques does not allow its use for these purposes. In addition, the available studies suggest that patients with PDAC in whom ctDNA is detected at the time of diagnosis have a poor prognosis and have a high chance of relapse after surgery, so it is advisable to develop more sensitive techniques that allow diagnosing tumors in earlier stages and provide patients with a better prognosis. It is possible that, with the development of ultrasensitive techniques, the joint use of different biomarkers and epigenetic marks, the sensitivity of LB will increase without losing specificity, and LB could be applied in PDAC screening.

The different LB methods have been shown to be a reliable biomarker in relation to the prognosis of patients with PDAC for both PFS and OS. Furthermore, its variations throughout treatment predict response or resistance to treatment several weeks in advance, so it could be used to guide treatment based on the evolution of the biomarker. In addition, the study of the characteristics of CTCs, ctDNA, exoDNA and miRNA can help us to better characterize the tumor and to identify potential therapeutic targets that facilitate the selection of treatment.

However, it is necessary to standardize and validate the methodology to be used in the different LB modalities. In addition, the usefulness of other LB modalities should be explored, such as lncRNAs or TEPs. On the other hand, the usefulness of less invasive sources of ctDNA such as urine or saliva needs to be further investigated.

Finally, it should be remembered that in order to apply LB to clinical practice, it is necessary to reduce costs, standardize protocols, and have data generated in the context of large-scale prospective clinical trials that confirm that the information provided contributes significantly to improving therapeutic results in PDAC patients.

## Figures and Tables

**Table 1:** Methods for isolation and analysis of liquid biopsy components in pancreatic cancer. Overview of the advantages and disadvantages of the described methods.

LB Component	Technique	Advantages	Disadvantages	Ref.
cfDNA	qPCR	Fast & Inexpensive High specificity	Lower sensitivity (0.1%) Detects only point mutations	[24,25,26,27,28,29,30]
	dPCR* (ddPCR, BEAMING)	High sensitivity (0.01%) & specificity	Detects only point mutations Expensive	
	NGS	High DNA input permits high throughput analysis and screen for unknown variants (WGS &WES) Can identify structural variants and copy number variations	Variable sensitivity (0.1% aprox.) Expensive	
Exosomes	Density-based isolation* (centrifugation)	Inexpensive Independent of marker expression	Time consuming High volume sample required Can damage exosomes Contaminated sample	[20,21,22,31,32,33,34,35]
	Size-based isolation	Fast & Inexpensive Independent of marker expression	Contaminated sample	
	Affinity-based isolation	High purity and specificity	Low sample yield	
	Commercial kits	Fast & Simple	Expensive	
CTCs	Immunoaffinity enrichment*	Positive enrichment: - Very specific - High capture efficiency & purity Negative enrichment: - Label-free CTCs obtained	Only one subpopulation captured Lower purity	[20,24,36,37,38,39,40,41,42,43,44,45,46,47,48,49,50]
	Physical methods (size & density)	Represent tumor heterogeneity Fast & Simple Less expensive Label-free CTCs obtained	Must be followed with immuno-labelling techniques to distinguish CTCs	
cfRNAs	RT-qPCR	Fast & Inexpensive High specificity	Low sensitivity in samples with low abundance cfRNA	[51]
	ddPCR*	Higher sensitivity & accuracy Lower sample volume required More reproducible than qPCR	Tedious assay optimization	

LB: liquid biopsy; qPCR: real-time quantitative-PCR; dPCR: digital-PCR; ddPCR: droplet-dPCR, NGS: next generation sequencing; RT-qPCR: quantitative reverse transcription PCR; WGS: whole genome sequencing; WES: whole exome sequencing. * Most used/gold standard.

**Table 2:** Potential liquid-biopsy clinical usefulness in early-diagnosis of pancreatic cancer.

	Study Technique	Specific/Relevant Molecular Findings	Stage	Nº Patients PDAC/Control	Sensitivity (%)	Specificity (%)	Ref.
**ctDNA**	ctDNA	*KRAS* mutant allele fraction > 5%	Early PDAC Metastatic	90/37 104/37	34 54	NR NR	[97]
		Mutations at codons 12, 13 and 61 of *KRAS*	Early PDAC	112	62	NR	[95]
		Mutations *KRAS* exon 2 (codons 12 and 13)	All stages	52/10	65	75	[94]
		*KRAS* MAFs	All stages	110/52	47	NR	[96]
		Mutations at codons 12, 13 of *KRAS*	Early PDAC	221/182	30	NR	[98]
		Mutations at codons 12, 13 and 61 of *KRAS*	Early PDAC	112/76	71	NR	[99]
**ctDNA combined with other serum tumor markers**	ctDNA+ CA19.9	Mutations *KRAS* exon 2	All stages	47/31	85-98	77-81	[100]
	ctDNA+ CA19.9	*KRAS* MAFs	All stages	110/52	47		[96]
	*KRAS* ctDNA + CEA + CA19.9 + HGF + osteopontin	Mutations at codons 12, 13 of *KRAS*	Early PDAC	221/182	64	99	[98]
**cfDNA methylome**	5-methylcytosine and 5-hydroxymethylcytosine	Global DNA hypomethylation	All stages	72/136	94	95	[101]
**CTCs**	CellSearch®	> 3 CTCs/mL	IPMN PDAC	21/19 8/19	33 73	NR	[102]
	CD45/CEP8/DAPI staining-FISH	CTC detection	PDAC	95/48	76	68	[103]
	Anti-EpCAM Portal-vein blood	Number of CTCs in 2 mL portal venous blood (if > 112 indicate hepatic metastasis)	Metastatic Resectable	17 43	100 58	NR	[104]
	Combined analysis CTC + exosomes	CTC detection and GPC1-positive-exosome detection	Early PDAC	22/28	100	80	[105]
**Exosomes**	*KRAS* mutations in exoDNA	Presence mutant *KRA*S in circulating exosome-derived DNA	Early PDAC Locally advanced Metastatic	33/54 15/54 20/54	67 80 85	NR NR NR	[106]
	GPC1-exosomes	CTC detection and GPC1-positive-exosome detection	Early PDAC	22/28	50	90	[105]
	miRNAs of exosomes	miRNAs in exosomes	All stages		Reported increased expression in PDAC	NR	[107,108,109,110]
**miRNAs**	miR-21, miR-25	miR-21, miR-25	All stages	303/760	75	93	[111]
	Meta-analysis	Presence of different miRNAs	All stages	4,326/4,277	79	74	[112]

ctDNA: circulating tumor DNA; PDAC: pancreatic ductal adenocarcinoma; NR: not reported, CTC: circulating tumor cells; miRNA: micro-RNA; HGF: hepatocyte-growth factor; CEP8: chromosome 8 centromere; FISH: fluorescence *in situ* hybridization; IPMN: intraductal papillary mucinous neoplasm; EpCAM:epithelial cell adhesion molecule.

**Table 3:** Liquid biopsy in pancreatic cancer ongoing clinical trials.

Trial	Trial Design	Trial Purpose	Study Population	*n*	Primary Endpoint	Technique	Ref.
DYNAMIC-Pancreas: ctDNA Analysis Informing Adjuvant Chemotherapy in Early Stage PDAC: A Multicenter Randomized Study	Phase II/III	Prognostic	PDAC locally advance treated with neoadjuvant chemotherapy and surgery	408	DFS	ctDNA	ACTRN12618 000335291
Mutation of *KRAS*, *CDKN2A*, *SMAD4* and *TP53* in PDAC	Role of Liquid Biopsy in Preoperative Diagnosis	Diagnostic	Non metastatic PDAC without any systemic metastatic spread at preoperative imaging	50	1-Presence of venous and/or arterial invasion 2-Early recurrence [<12 months from resection], local or systemic recurrence after resection	*KRAS*, *CDKN2A*, *SMAD4 * and *TP53* mutation on circulating cfDNA	NCT03524677
Prognostic Role of ctDNA in Resectable PDAC (PROJECTION)	Comparison of DFS of patients with preoperative presence of ctDNA (Group A) and absence of ctDNA (Group B)	Diagnostic Prognostic	Resectable PDAC	200	To determine the stage, the remission or the progression of PDAC	Collected prior of surgery and within 14 days before start of adjuvant chemotherapy.	NCT04246203
Detection of High Expression Levels of EMT-Transcription Factor mRNAs in Patients with PDAC and Their Diagnostic Potential	Case control	Diagnostic	Case: Cases Subjects affected by PDAC Control: Healthy Subject enrolled following colon cancer screening via colonoscopy	850	DFS	Detection and quantification of EMT-transcription factor mRNA levels in blood	NCT04323917
Verification of Predictive Biomarkers for PDAC Treatment Using Multicenter Liquid Biopsy	Observational	Diagnostic	Subjects affected by PDAC	662	Clinical applicability	1-Quantification and monitoring of *KRAS* mutations Using ddPCR in ctDNA 2-Discovery of biomarkers through ctDNA panel	NCT04241367
Circulating Extracellular Exosomal Small RNA as Potential Biomarker for Human PDAC	Cohort-prospective	Diagnostic Early-detection	PDAC and other pancreatic lesions	102	Sensitivity and specificity of exo-sRNA analysis	exo-sRNA	NCT04636788
Diagnostic Accuracy of CTCs and Onco-exosome Quantification in the Diagnosis of PDAC-PANC-CTC (PANC-CTC)	Cohort-prospective	Diagnostic	PDAC	52	Sensitivity and diagnostic application of CTC detection	CTC	NCT03032913
PRIMUS002: Looking at 2 Neo-adjuvant Treatment Regimens for Resectable and Borderline Resectable PDAC	Phase II non-randomized	Prognostic	-FOLFOX-A (FOLFOX + Nab-paclitaxel) -AG: Nabpaclitaxel-Gemcitabie	278	Time to progression	ctDNA	NCT04176952
Tumor Markers, Liquid Biopsies, and Patient Reported Outcomes in Metastatic Colorectal, Pancreas, Biliary, and Esophagogastric Cancers	Observational multicohort	Prognostic	Gastrointestinal Cancer	600	RECIST response	CEA, CA19.9 and ctDNA	NCT04776837
PLATON-Platform for Analyzing Targetable Tumor Mutations (Pilot-study)	Observational multicohort	Diagnostic	Gastrointestinal Cancer	200	Relative frequency of targetable mutations	FoundationOne^®^CDx and FoundationOne^®^Liquid	NCT04484636
ctDNA in Pancreatic Cancer	Prospective observational	Diagnostic	Resectable pancreatic cancer	100	Analysis of Factors Related to PDAC Recurrence Using ctDNA	ctDNA	NCT02934984
A Study of Blood Based Biomarkers for Pancreas Adenocarcinoma	Prospective observational	Diagnostic Early-detection	PDAC and benign pancreatic disease	750	Sensitivity for early diagnosis	Proteins and proteases, functional DNA repair assays, exosomes, stromal elements, cRNAs and ctDNA	NCT 03334708
Blood Markers of Early Pancreas Cancer	Prospective observational	Early-detection	New onset diabetes, high risk pre-diabetes Pancreatic cystic neoplasms and pancreatitis Familial risk	1250	Sensitivity for early diagnosis	cfDNA	NCT03568630
Nalirinox Neo-pancreas *RAS* Mut ctDNA Study	Phase II	Prognostic	Patients with Resectable PDAC Treated with Neoadjuvant NALIRINOX	20	Monitoring response	*KRAS* ctDNA	NCT04010552

ctDNA: circulating tumor DNA; cfDNA: cell free DNA; cRNAs: circular RNAs; PDAC: pancreatic ductal adenocarcinoma; DFS: disease-free survival; CTCs: circulating tumor cells; EMT: epithelial-mesenchymal transition.

**Table 4:** Prognostic performance of liquid biopsy in PDAC.

Tumor Stage	Nº Patients Total ctDNA + ctDNA-	Results	Ref.
Resectable	59 29 30	RFS 8mo if pre-surgery ctDNA + vs. 19mo if ctDNA- (*p* < 0.01)	[135]
Resectable	37 23 14	RFS 10.3mo if pre-surgery ctDNA + vs. RFS not reached (*p* = 0.002)	[95]
Resectable	34 14 * 20 *	*ExoDNA KRAS* MAF peak of ≥1% after treatment is associated with tumor progression	[97]
Advanced/metastatic	104 50 54	OS 6.5mo if ctDNA + vs. 19mo if ctDNA- (*p* < 0.001)	[154]
Advanced/metastatic	55 42 13	OS 2.5mo if ctDNA + with copy number gain vs. 5.5mo without copy number gain vs. 10.6mo if ctDNA- (*p* < 0.001)	[155]
Metastatic	61 47 14	OS 5.6mo if ctDNA + vs. 12.4mo if ctDNA- (*p* < 0.001)	[156]
Metastatic	102 70 32	OS 8.6mo if ctDNA + vs. 14.6mo if ctDNA- (*p* < 0.02) PFS 3.5mo if ctDNA + vs. 10.7mo if ctDNA- (*p* < 0.02)	[97]
Any stage	77 60 17	*KRAS* MAF peak of <0.415% is associated with longer PFS and OS	[157]
Advanced/metastatic	54 36 18	Decrease in *KRAS* ctDNA levels during chemotherapy (d14) is an early indicator of response to treatment	[158]
Metastatic	113 77 36	Early change in ctDNA levels (d28) was correlated with ORR, PFS and OS	[159]
Advanced/metastatic	38 17 21	The dynamics of total cfDNA concentration correlated with tumor burden following chemotherapy	[117]
Metastatic	188 65 123	OS 4.7mo if ctDNA + vs. 6mo if ctDNA- (*p* = 0.015)	[160]

ctDNA: circulating tumor DNA; MAF: mutant allele fraction; RFS: recurrence-free survival; OS: overall-survival; ORR: objective response rate; * ExoDNA.
